# ImtRDB: a database and software for mitochondrial imperfect interspersed repeats annotation

**DOI:** 10.1186/s12864-019-5536-1

**Published:** 2019-05-08

**Authors:** Viktor N. Shamanskiy, Valeria N. Timonina, Konstantin Yu. Popadin, Konstantin V. Gunbin

**Affiliations:** 1 Center for Mitochondrial Functional Genomics, School of Life Science, Immanuel Kant Baltic Federal University, Kaliningrad, Russia; 2 Center for Integrative Genomics, University of Lausanne, Lausanne, Switzerland; 3 Swiss Institute of Bioinformatics, Lausanne, Switzerland; 4 Center of Brain Neurobiology and Neurogenetics, Institute of Cytology and Genetics SB RAS, Novosibirsk, Russia

**Keywords:** mtDNA, Imperfect repeats, Database, Selection on dinucleotides

## Abstract

**Background:**

Mitochondria is a powerhouse of all eukaryotic cells that have its own circular DNA (mtDNA) encoding various RNAs and proteins. Somatic perturbations of mtDNA are accumulating with age thus it is of great importance to uncover the main sources of mtDNA instability. Recent analyses demonstrated that somatic mtDNA deletions depend on imperfect repeats of various nature between distant mtDNA segments. However, till now there are no comprehensive databases annotating all types of imperfect repeats in numerous species with sequenced complete mitochondrial genome as well as there are no algorithms capable to call all types of imperfect repeats in circular mtDNA.

**Results:**

We implemented naïve algorithm of pattern recognition by analogy to standard dot-plot construction procedures allowing us to find both perfect and imperfect repeats of four main types: direct, inverted, mirror and complementary. Our algorithm is adapted to specific characteristics of mtDNA such as circularity and an excess of short repeats - it calls imperfect repeats starting from the length of 10 b.p. We constructed interactive web available database ImtRDB depositing perfect and imperfect repeats positions in mtDNAs of more than 3500 Vertebrate species. Additional tools, such as visualization of repeats within a genome, comparison of repeat densities among different genomes and a possibility to download all results make this database useful for many biologists. Our first analyses of the database demonstrated that mtDNA imperfect repeats (i) are usually short; (ii) associated with unfolded DNA structures; (iii) four types of repeats positively correlate with each other forming two equivalent pairs: direct and mirror versus inverted and complementary, with identical nucleotide content and similar distribution between species; (iv) abundance of repeats is negatively associated with GC content; (v) dinucleotides GC versus CG are overrepresented on light chain of mtDNA covered by repeats.

**Conclusions:**

ImtRDB is available at http://bioinfodbs.kantiana.ru/ImtRDB/. It is accompanied by the software calling all types of interspersed repeats with different level of degeneracy in circular DNA. This database and software can become a very useful tool in various areas of mitochondrial and chloroplast DNA research.

## Background

Till now the mtDNA determinants of longevity, i.e. germline mtDNA variants which cause aging and thus correlate with longevity, are poorly known [1]. Long-lived mammals have increased GC content [2], decreased amount of direct (both perfect and imperfect) and inverted repeats [3–7], and shorter length of mtDNA [5]. Probably, some of these correlations are driven by the decreased somatic mutation rate in long-lived mammals (GC-rich DNA with decreased amount of repeats is expected to be more stable in somatic tissues), however, still there is a lack of understanding of the mechanisms of these correlations: which type of repeats better correlate with longevity - inverted or direct, perfect or imperfect? Why? For example, originally, it has been shown that perfect (with identical sequences of both arms) direct repeats mark somatic deletion breakpoints and thus might be important for origin of deletion [8, 9]. Later, the perfect direct repeats as the main determinants of somatic deletions were extended to long imperfect repeats (duplexes) [10]. These observations of non-uniform distribution of somatic deletions were supported by comparative-species analyses where negative correlation between species-specific lifespan and abundance of perfect [5] and imperfect direct repeats [7] has been shown. Interestingly, all previous works focused on major arc of mtDNA, but if authors take into account the whole mtDNA excluding D-loop (i.e. major and minor arcs together), the negative correlation disappeared [11]. Additionally, it has been shown that not only direct, but also inverted repeats negatively correlate with animal lifespan [3] probably by inducing mtDNA inversions and mitochondrial genome instability during mtDNA replication cycle [6]. Recent analyses of the distribution of somatic deletions along the human mtDNA demonstrated that mtDNA deletions may also depend on imperfect repeats of various nature (i.e. direct, inverted, mirror, complementary) between distant mtDNA segments [10, 12, 13] and also on various non-B-DNA structures (such as G-quadruplexes) [10, 13, 14], opening a possibility of existence of others, not yet described, mtDNA components of longevity. Altogether, till now there is no common and well established model, explaining causative effects of mtDNA repeats on animal lifespan. To answer this question we need to construct a database with all types of nucleotide repeats called by the same algorithm for each species with sequenced complete mitochondrial genome.

There are four main types of interspersed repeats, different from the point of view of location of their arms (the same strand: direct and mirror or different strands: inverted and complementary) and direction (the same direction: direct and complementary; opposite direction: mirror and inverted); additionally each repeat type can be characterized by its level of degradation (perfect and imperfect). Currently existed databases consider only limited number of repeat types (only direct for example) and / or their level of the degeneration (only perfect for example). The merging of such specialized databases together is non-rationale because of different algorithms, used to call various repeat types, as well as different subsets of analyzed species. Thus, here we derive our own algorithm to call four types of interspersed repeats and our integral database storing these mtDNA repeats for all chordata species with sequenced complete mtDNA.

In our algorithm and database we analyze all four types of interspersed repeats and don’t consider short tandem repeats or microsatellites. Annotation of the short tandem repeats require completely different approaches that is against the main idea of our database - to call all repeat types using the same base algorithm. Nevertheless below we review briefly main algorithms and databases of the short tandem repeats. Mitochondrial microsatellite instability is associated with various diseases, including human cancers [15], such as colorectal [16], endometrial [17] and breast [18] cancer. There are several comprehensive databases depositing and annotating microsatellites, for example, FishMicrosat [19], including two databases specialized for mtDNA microsatellites: ChloroMitoSSRDB includes information about perfect, imperfect and complex mitochondrial microsatellites of animals [20], MitoSatPlant describes mitochondrial microsatellites of plants [21]. Correspondingly, there are several software tools intended to discover and annotate microsatellites. Some of them can identify only perfect microsatellites (SSRIT [22], Poly [23], TROLL [24], GMATo [25], GMATA [26]) while others can identify also imperfect microsatellites (TRF [27] and pSTR Finder [28], Sputnik [29], Star [30], G-IMEx [31], mreps [32], TandemSWAN [33], SciRoKo [34], Dot2Dot [35], ProGeRF [36], BWtrs [37], T-REKS [38], XSTREAM [39], SSR Locator [40]).

Now, to the best knowledge of the authors, there are no databases depositing and annotating both perfect and imperfect mtDNA interspersed repeats of any length and nature. For example, two most popular and comprehensive databases depositing interspersed repeats in organellar and nuclear genomes are focused mainly on repeats of transposon and tRNA nature (RepBase [41], Dfam [42]).

A number of computing algorithms have been developed to call imperfect interspersed repeats of all four classes. They can be roughly divided in four groups by the strategy of repeats discovery: (1) algorithms based on the local pairwise alignment (using, for example, heuristic search by BLAST [43], or exact search by PALS [44] or constructing suffix trees mediated by maximal unique match (MUM) finding implemented in MUMmer [45]); (2) tools using dot-matrix analysis; (3) algorithms that are based on k-mer overrepresentation analysis, and (4) algorithms searching for periodicities using Fourier transforms. The most famous software tool for repeats identification based on the precompiled repeat database is RepeatMasker [46]. Repeats discovery tools based on local pairwise alignment and/or self-alignment in comparison with k-mer (or l-mer, N-mer i.e. short substrings of nucleotides) overrepresentation analysis tools could potentially allow more accurate identification of short copy repeat sequences and more accurate recognition of the flanking regions. Local pairwise alignment preliminary step is required, for example, for RECON [47], REPET [48], PRAP [49] and PILER [50] calculations; while for RepEx [51] preliminary identification of MUMs is required. Dot-matrix visualization and analyses are implemented for example in DOTTER [52], Adplot [53], Gepard [54], JDotter [55], PLOTREP [56], r2cat [57], D-GENIES [58]. Dot matrix analysis usually required visual inspection of resulted graphics relating with various drawbacks in identification of repeat flanking regions due to large window size. Identification of repeats could be done using adjusted k-mers frequency as seeds, and greedily (by naïve algorithm or constructing suffix arrays/trees or by identification of elementary repeats) extension of each seed to a longer consensus sequence. This approach has been implemented for example in RepeatScout [59], REPuter [60], SPADE [61], WindowMasker [62], Vmatch [63], phRAIDER [64]. Fourier transforms based algorithms are implemented for example in nucleotide-based software Spectral Repeat Finder [65] and SBARS [66] and dinucleotide-based DNADU [67]. It is of note that Fourier power spectrum may not characterize repeats precisely due to the inability to identify repetitive pattern, copy number and the level of degeneration.

All above described algorithms work with linear DNA. However, the mitochondrial DNA is circular and it is important to consider this property in repeats discovery. The only program working with circular molecules is RepeatAround [68], however, it does not allow to call imperfect repeats.

Another peculiarity of mtDNA repeats is their quite short length, with majority of them shorter than 20 bases [1, 3, 5–7, 10, 20, 21]. Also, from the plethora of experimental DNAseq and/or RNAseq data as well as from the features of miRNA/mRNA interaction [69–77] it is known that there is a limit on the minimal length of DNA or RNA stretches that are useful for base-pairing (for example, perfect base pairing of 7–8 nucleotides of miRNA seed region is required for proper miRNA/mRNA interaction [69]). Thus, it is important to design an algorithm focused on short repeats - not all existed algorithms are able to work with imperfect repeats as short as 10 b.p.

**Fig. 1:**
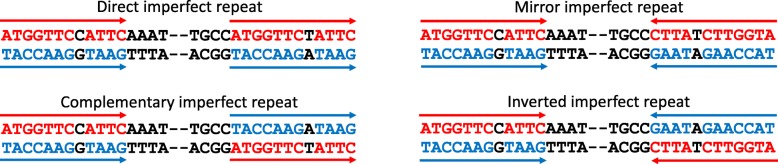
Four interspersed repeat types. Colors mark the repeated nucleotide pattern, arrows indicate the pattern direction

Here we describe our database ImtRDB (http://bioinfodbs.kantiana.ru/ImtRDB/) where we store and analyse mtDNA repeats annotated by our algorithm in all chordata species with sequenced complete mitochondrial genome. This database is focused on interspersed repeats of four basic classes (Fig. 1) with different level of degeneration (perfect and non perfect). In order to call these repeats we implemented simple dot-matrix-based algorithm, which fits two important mitochondrial properties: circularity and an excess of short repeats. Using the database, we demonstrated strong positive correlations between the abundance of direct and mirror repeats as well as between inverted and complementary repeats. We note that these pairs (direct and mirror; inverted and complementary) have identical nucleotide content of the repeat arm (see Fig. 1: first arm of the direct repeat has two ‘A’, five ‘T’, two ‘G’ and two ‘C’; the same content on the same strand we will observe in case of the second arm of the direct repeat as well as on the second arm of the mirror repeat) and thus they can be considered as equivalent repeats, i.e. if we assume the same rate of origin (mutagenesis) as well as the same rate of decay (selection coefficients) we expect to see equal numbers of equivalent repeats. We think that the equality of the equivalent repeats is a useful null hypothesis, which can be tested in the future. Additionally we confirmed deficit of C and G nucleotides in repeat-rich genomes and demonstrated previously unknown excess of GC over CG dinucleotides in the light chain of mtDNA, covered by repeats. We also confirmed that mtDNA repeats are usually short and associated with unfolded DNA structures. Our database as well as our first several observations will facilitate future discoveries of the functional roles of mtDNA repeats.

## Construction and content

### Repeats searching algorithm

**Fig. 2:**
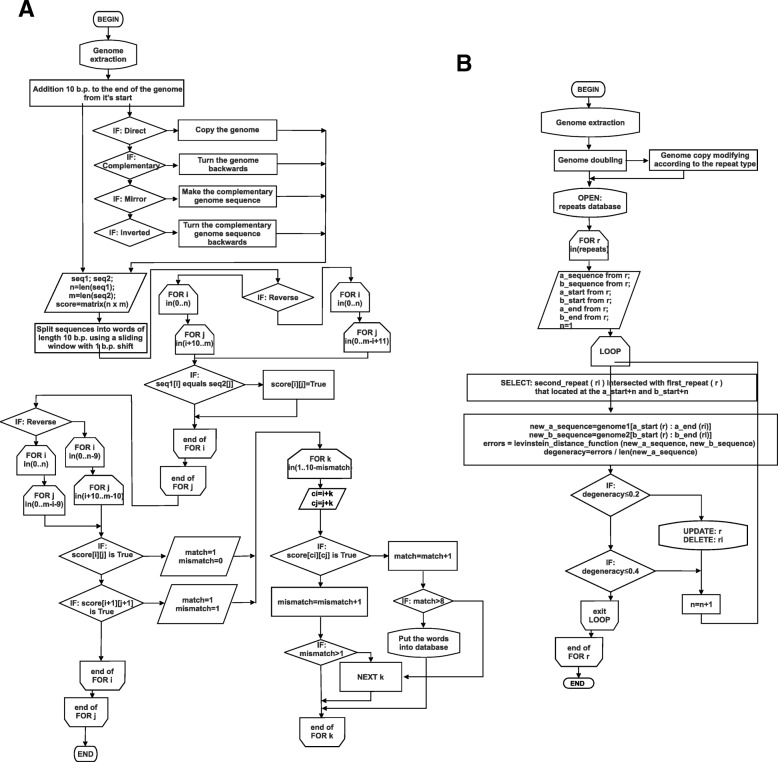
Block scheme of repeats searching algorithm. A - recognition of similar short nucleotide patterns; B - short patterns merging

We implemented in Python naïve algorithm of pattern recognition by analogy to standard dot-plot construction procedures. The algorithm consists of two stages: (I) recognition of similar short nucleotide patterns and (II) short patterns merging (Fig. 2).

Recognition of similar short nucleotide patterns is based on predefined sliding window of 10 b.p. length and maximum 10% degeneracy of this length. To consider circularity of mtDNA at the {*step 1*} of the stage I we copy 10 nucleotides from mitochondrial genome start position (as defined in genbank file) to the end of genome, thus elongating the genome by 10 b.p.. For this elongated (or main) mtDNA {*step 2*} we generated four supplementary sequences: identical to this sequence (a copy), the complementary, the reversed and reverse-complement. The main procedure of similar short nucleotide patterns searching {*step 3*} is conducted as sliding window analysis on the half of L*L square symmetrical ‘dot-plot’ matrix, where *L* is mtDNA length + 10b.p.. We used four supplementary sequences in ‘dot-plot’ matrix to find different types of repeats: we used copy of the main sequence for direct repeats finding, complementary sequence used for everted (or complementary) repeats identification, reversed sequence used for mirror (or centrally symmetric) repeat detection, and reverse-complement sequence used for inverted repeat detection. If repeated pattern was found {*step 4*}, we fixed the coordinates of both (query and target) sequences in genome as a SQLite database entry containing locations of two genome segments. Overall the computation time complexity of recognition of similar short nucleotide patterns stage is O((m-10)^4^), where m is the size of genome under analysis.

In order to find repeat patterns longer than 10 b.p., we screen iteratively all short-repeats (obtained on stage I) for their intersection. We confirm intersection of two short repeats if repeated sequences of both repeats in two or more genome locations have equal and collinear (in terms of repeats type) displacement {*step 1*} with respect to each other. For example, consider two short repeats, A and B, each representing by two arms (monomers) - query and target. For direct and complementary (or everted) repeats (the pairs of target_A and query_A monomers) we searched in SQLite database the intersected repeats (pairs of target_B and query_B monomers) shifted in genome coordinates by the equal b.p. length toward to the end or start of genome. For inverted and mirror repeat genome positions we searched in SQLite database the intersected positions of query_B and target_B monomers shifted by the equal b.p. length inward or outward relative to the minimal genome segment located between query_A and target_A monomers in circular mtDNA. As a result of each round of short-repeats merging we elongate (change genome coordinates) one repeat in SQLite database and delete the other that intersected with this one. We do this if and only if the {*step 2*} resulted merged repeat has not more than 20% of degeneracy. We iterated *step 1* and *step 2* of short patterns merging stage until there are no new merged repeats generated. It is of note that to consider mtDNA circularity the short patterns merging stage was based on duplicated genome generated by concatenation of two genome sequences. Overall the computation time complexity of short patterns merging stage is O(2m^2^), where m is the number of simple repeats found on the first stage.

### Web-interface and database construction

We used NCBI E-utilities [78] for retrieval 4694 Vertebrate mitochondrial genome GenBank files (as listed on 2018 Mar 27 on NCBI Organelle Genome Resources).

In order to structurize the data in database, make them interactive and freely available we made web-available resource (http://bioinfodbs.kantiana.ru/ImtRDB/). To do it we used Apache web-server, MySQL 5, Perl 5.24 (CGI module), HTML5, and JavaScript for web-pages dynamical generation. We used jBrowse [79, 80] for interactive visualization of mitochondrial genomes and several repeat tracks. MySQL database is very simple, it consists of 3 relational tables: a table containing species taxonomy extracted from NCBI Taxonomy, which is used for species searching by taxonomy; a table linking NCBI ID of mtDNA with species names; and a table containing the results of correlation analyses (Pearson R and Spearman Rho) between repeat densities and the repeats physico-chemical features, which are used for species-specific correlation pages generation (see details below).

### Statistical analyses of repeats physico-chemical features and content

**Fig. 3:**
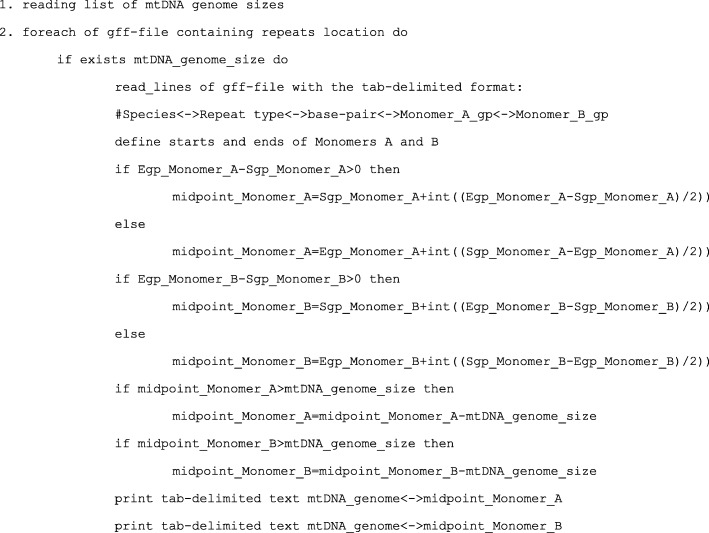
Pseudocode of Perl script for calculating midpoint position for each repeat monomer. Abbreviations: gp, genome position of nucleotide; Sgp, start genome position of repeat monomer; Egp, end genome position of repeat monomer

In order to characterise the distribution of repeats along each mitochondrial genome, we correlated density of repeats in a given region with several physico-chemical features of the region. First, we calculated the midpoint position for each arm (monomer) of each repeat as an integer of (start_position+(end_position-start_position)/2) (Fig. 3). There are at least two arms (monomers) in each repeat, therefore each repeat is characterized by at least two midpoint positions dispersed in the mitochondrial genome. Second, for each monomer sequence (arm) of each repeat, we calculated various physico-chemical features (Emboss package v. 6.6 [81]) and assigned these values to the midpoint position of the arm. We used the following Emboss programs and corresponding features: (1) btwisted (for calculating total stacking energy; average stacking energy per dinucleotide; total turns; average bases per turn and total twist in degrees); (2) dan under ‘-thermo’ option (for calculating GC Content, %; melting temperature of repeat regions base-pairing; change in Gibbs free energy, Enthalpy and Entropy in repeat regions base-pairing); (3) compseq (for calculating 16 fractions of dinucleotides). Third, we correlated the number of repeats in midpoints (the density of repeat monomers having the same midpoint) with average physico-chemical features assigned to each midpoint at the step two. All statistical analyses were done in R v. 3.4.1.

### Visualization of repeats

The most straightforward way to present the distribution of repeats in mitochondrial genome would be to draw arks between each pair of arms. However, this way of visualization is difficult to perceive due to high number of repeats in each genome and high number of arms in each repeat (two arms - one arc, three arms - three arcs, four arms - six arcs etc). In order to minimize the number of arcs to draw we focused on three measures (minimal and maximal distance between arms of a given repeat and distance between arms with maximal similarity) and implemented a probabilistic procedure of the linking of the repeat arms so that the closest arms will have high chances to be linked by an arc. The probability of repeat monomers (arms) linking by ark is given by the exponential probability distribution with a mean of the distribution equals to 1/16 of mitochondrial genome length. We generated these linking probabilities on a section from 0 to 1/2 of mitochondrial genome length (due to genome circularity two genome points distant by the half of genome length are the most distant points). So, if the distance between complementary repeat regions is significantly higher than 1/16 of mitochondrial genome than the probability of complementary regions linking by ark tends to be zero.

## Utility and discussion

### Testing the repeats searching algorithm

Our algorithm is intended to find both perfect as well as imperfect repeats. We choose minimum scanning repeat length equals to 10 bases with maximum one mismatch. This length and degeneracy threshold was selected because on average DNA has 10 bases in helix turn and the minimum biologically meaningful pairing is about eight-ten bases long (for example, canonical seed-matched sites of miRNA-mRNA pairing is 7–8 bases long [69], however base-pairing beyond seed region is necessary for miRNA function [70]; randomly amplified polymorphic DNA technique optimal primers length is ten bases [71–73]; minimal match length for the meaningful intensity of probe hybridization in the presence of DNA with perfect match is about 10 bases [74]). For longer repeat lengths, we considered maximum 20% of degeneracy that is allowed to form highly stable structures despite the presence of unpaired bases. We allowed only non-tandem mismatches because of 1) the average length of previously known mitochondrial imperfect repeats is not more than 20 bases [1, 3, 5–7, 10, 20, 21]; 2) thermodynamics of duplex formation in the case of interspersed mismatches is additive, linear and well-established [74, 75]; 3) the dependence of the duplex stability of tandem mismatch on the identity, length and context-specificity of the flanking base pairs [76]. Additionally, we keep off indels due to the same reasons (context-specific dependence of the bulges stability) [74, 77]. These simplifications are biologically exceptionally meaningful due to short nature of mitochondrial imperfect repeats found in this study (on average 12 bases).

We tested our algorithm by comparing with the published ones. For this purpose, we chose two well described mitochondrial genomes – *Homo sapiens* genome (NC_012920) and *Mus musculus* (AY172335) genome. We selected three algorithms for comparison (Table 1): state-of-the-art sought-after algorithm Vmatch [63], RepeatAround intended to circular DNA analysis [68], and universal RepEx algorithm based on the maximal unique matches [51].

**Table 1:** Comparison of our repeats searching algorithm with early published ones

**Genome**	**Repeat type**	**Our algorithm** ^**1**^	**Vmatch imperfect** ^2^	**Vmatchperfect** ^3^	**RepEx** ^**4**^	**Repeat-Around** ^**5**^
***Homo sapiens***	direct	6304 (6135 impf. 169 pf.)	2507 (2507 impf.) 1358 common	320 pf., common	–	333 pf., t.l.
	complimentary	1694 (1654 impf. 40 pf.)	–	–	70 pf., common	7 pf., t.l.
	mirror	5416 (5295 impf. 121 pf.)	–	–	252 pf., common	83 pf., t.l.
	inverted	1939 (1868 impf. 71 pf.)	1984 (1974 impf., 10 pf.) 1937 common	127 pf., common	110 pf., common	35 pf., t.l.
***Mus musculus***	direct	6765 (6594 impf. 171 pf.)	2543 (2543 impf.) 1325 common	308 pf., common	–	323 pf., t.l.
	complimentary	3580 (3511 impf. 69 pf.)	–	–	143 pf., common	50 pf., t.l.
	mirror	6029 (5871 impf. 158 pf.)	–	–	286 pf., common	97 pf., t.l.
	inverted	3873 (3772 impf. 101 pf.)	3947 (3929 impf. 18 pf.) 3853 common	195 pf., common	179 pf., common	63 pf., t.l.

^1^impf. and pf. denotes imperfect and perfect repeats, respectively

^2^Vmatch run options for imperfect repeats finding: 1) for direct repeat length 10 the allowed hamming distance 1 (90% identity), for direct repeat lengths from 11 to 100 the allowed hamming distance is integer of L/5, where L is the repeats length (80% identity) ‘– supermax’ option was used for all repeat lengths, 2) for inverted repeat lengths with lengths from 10 to 100 the allowed hamming distance is varied from 1 to 10 for each length (minimum identity seeks from 90 to 80% with repeat length growth). After the repeats retrieval, all doubles were disregarded as well as inner repeats (or sub-repeats) with a smaller length than searched; all intersected repeats were merged into longer ones. “Common” denotes common repeat patterns between our algorithm and previous three algorithm

^3^Vmatch run options for perfect repeats finding: ‘-identity 100’ option, repeat lengths from 10 to 100 for direct and inverted repeats

^4^RepEx run options: minimum length 10; spacer intervals greater than 0; sequence degeneracy allowed

^5^RepeatAround run options: repeat lengths from 10 to 256. RepeatAround “t.l.” denotes typical locations or, in other words, locations matched graphically by hand with repeat positions found by our algorithm (see details in text)

First of all, Table 1 shows that the vast majority of repeats in selected mtDNAs are imperfect (compare the numbers of repeats found by RepEx and RepeatAround with those found by our algorithm and Vmatch). Therefore, perfect repeat occurrence might be under negative selection, that is consistent with previous data [1, 3, 5–7, 10, 20, 21].

Second, data in Table 1 shows that all perfect repeats found by Vmatch (*Homo sapiens*: 447, *Mus musculus*: 503) and RepEx (*Homo sapiens*: 432, *Mus musculus*: 608) have been found also by our algorithm. RepeatAround is old Windows software that did not run correctly on the contemporary Windows 7 and 10 operation systems as well as on Windows XP and Windows 2000  virtual machines (“Unable to register controls!” error occurred while RepeatAround starting). Thus, we were able to compare results of RepeatAround with our results only by hand (visually). Nonetheless, all randomly selected for visual inspection repeats called by RepeatAround were also called by our algorithm. Thus, we conclude that our algorithm recovers all perfect repeats.

**Fig. 4:**
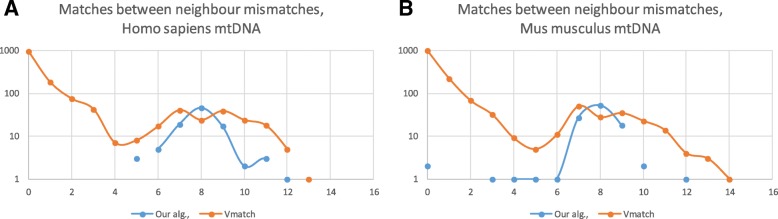
Number of nucleotides between neighbor mismatches in imperfect repeats found by our algorithm and Vmatch in (A) *Homo sapiens* mtDNA and (B) *Mus musculus* mtDNA

Despite the ability to find accurately perfect repeats our algorithm is computationally harder comparing to other algorithms, for instance, to Vmatch algorithm [63]. Therefore, in order to define our algorithm advantages, it is necessary to compare the results of our algorithm with Vmatch ones. The comparison of two table columns ‘Our algorithm’ and ‘Vmatch imperfect’ clearly demonstrates that only a small fraction of repeats are found by both algorithms. In order to explain this discrepancy we compared the number of nucleotides between neighbour mismatches in all imperfect repeats found by Vmatch and our algorithm. We observed that Vmatch found significantly higher number of imperfect repeats with tandem substitutions (left part of the plot) while our algorithm selected such cases out (Fig. 4). As we described before the avoiding of the tandem mismatches is beneficial for our algorithm since it allows us to filter out unstable repeats with long (> 1) tandem mismatches. It is also of importance that Vmatch did not found vast majority of 10 b.p. repeats with single mismatch and longer repeats with dispersed mismatches while our algorithm effectively found such repeats.

### Database statistics and user interface

**Fig. 5:**
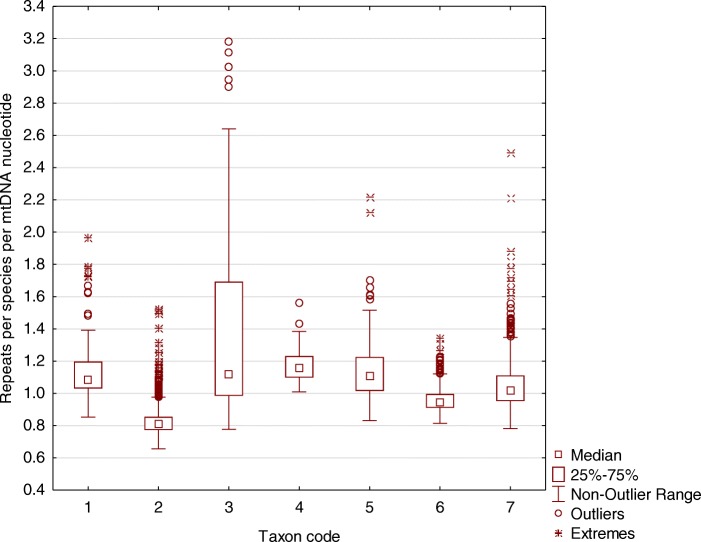
The number of all four types of repeats normalized by mtDNA lengths of each species. Taxon codes: 1, Chondrichthyes; 2, Actinopterygii; 3, Amphibia; 4, Testudines; 5, Squamata; 6, Aves; 7, Mammalia

The ImtRDB database, to the best knowledge of the authors, is the first database depositing interspersed mitochondrial imperfect repeats. The ImtRDB database now has 4694 entries (Vertebrate mitochondrial genomes), 3716 of them have been processed (annotated). The list of all analyzed species can be viewed using button ‘You can list all annotated mtDNAs by taxonomic *Families* or *Classes*.’ located at the top of the page. In order to compare the number of repeats between species with different genome size, we normalized number of repeats by genome length and got the number of repeats per nucleotide. This number of repeats per nucleotide in seven taxa of Vertebrata is shown on Fig. 5.

Using button ‘species searching’ or ‘all species listing’ it is possible to get a table containing three columns: species name, NCBI taxonomy and repeats GFF file available for downloading. In case of ‘species searching’ there are checkboxes in the third column, which allow user to compare repeat numbers per nucleotide between selected species (now maximum 4 species can be compared) by multiple alignment of mtDNAs (using MUSCLE v3.8.31). This comparison is possible to run clicking ‘submit’ button on the bottom of the ‘species searching’ page. Clicking on species name user can access species-specific mtDNA genome data that are integrated upon jBrowse user interface. Using REFSEQ NC ID button, located in the third table column user can download raw GFF-file containing repeat genome positions. Additionally, for each annotated species correlations between repeats density and physico-chemical features can be viewed by clicking on the plot icon located near the species name in the first column of the table.

The user interface of species-specific data is based on jBrowse [79, 80]. The benefits of jBrowse user interface is well-known interface simplicity, interactivity and usability. For instance, user can easily select any track for visual inspection, zoom in and zoom out track data and slide along mtDNA genome; clicking on ‘wig formatted’ track in the main jBrowse window user can download all or selected track data in text format, for example, GFF format. For example, if user interested in detection of a species-specific regions with high number of repeats (of any kind) it is possible to download “Repeat midpoints density per nucleotide” or “Repeats density per nucleotide” tracks as a text files and made simple Z-test using, for example, R computations. Each annotated mitochondrial genome has 33 jBrowse features. These are genes locations and descriptions as in genbank file; repeats density per nucleotide; repeat midpoints density per nucleotide; 16 average fractions of dinucleotides mapped on repeat midpoint positions in mtDNA; average GC percent of repeats mapped to repeat midpoints; average melting temperature of repeat regions base-pairing mapped to repeat midpoints; average change in Gibbs free energy, Enthalpy and Entropy in repeat regions base-pairing, multiplied by − 1 and mapped to Repeat midpoints; average total Stacking energy and Stacking energy per dinucleotide in repeat regions base-pairing, multiplied by − 1 and mapped to repeat midpoints; average total turns and bases per turn in repeat regions base-pairing helix, mapped to repeat midpoints; average total twist in repeat regions base-pairing, in degrees, mapped to repeat midpoints; minimal and maximal distance between repeat arms (or monomers); distance between repeat arms with maximal complementarity. Unprocessed genomes have only one feature, the genes locations and descriptions.

### Examples of database usage

**Fig. 6:**
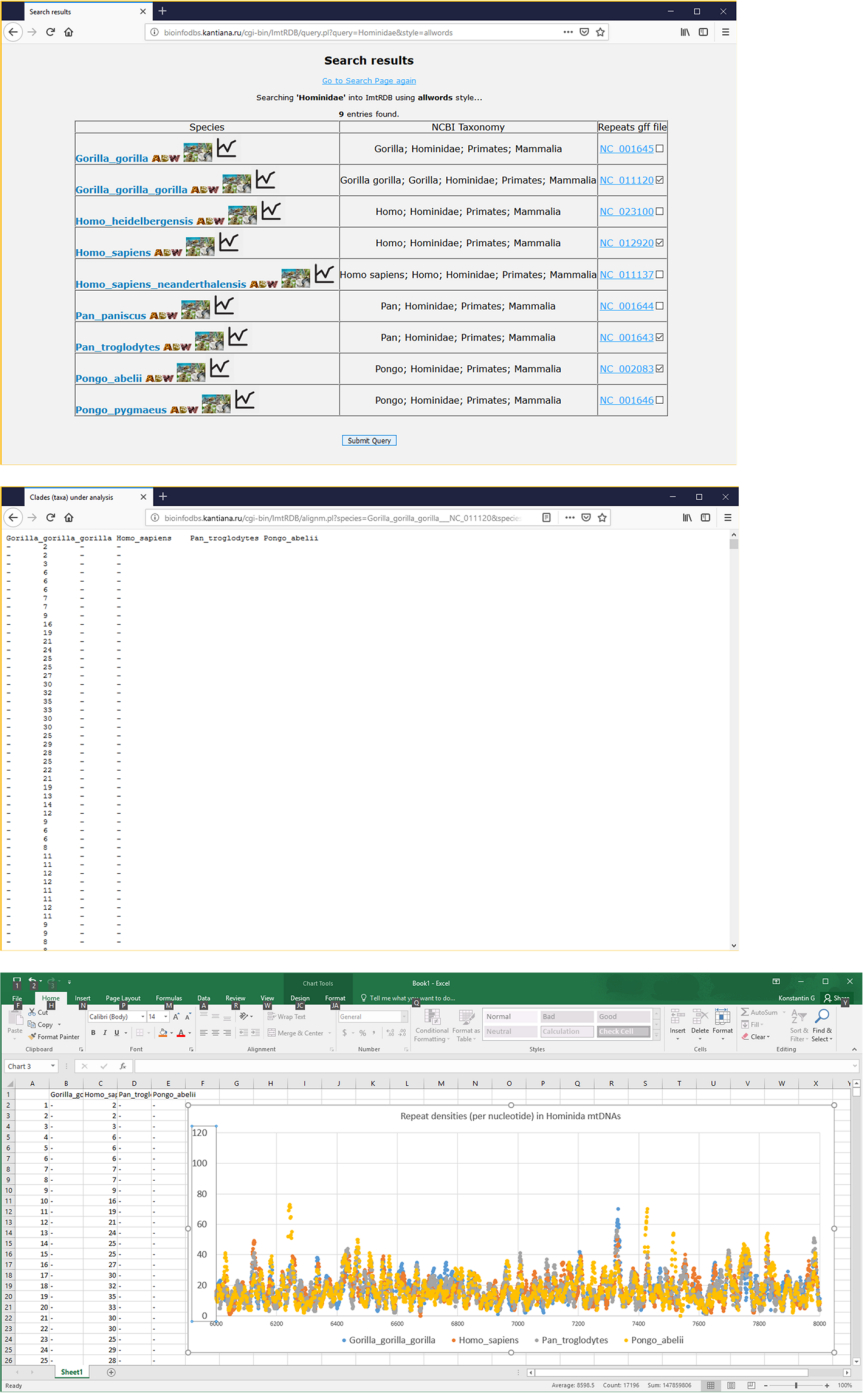
The analysis of repeat densities evolution in Hominidae. Upper screenshot shows ‘Search results’ page with selected mtDNAs for subsequent comparative analysis. Middle screenshot demonstrate the resulted tab-delimited table containing the alignment of repeat densities (per nucleotide). Bottom screenshot shows the plot generated in MS Excel based on tab-delimited table described in the middle screenshot

**Fig. 7:**
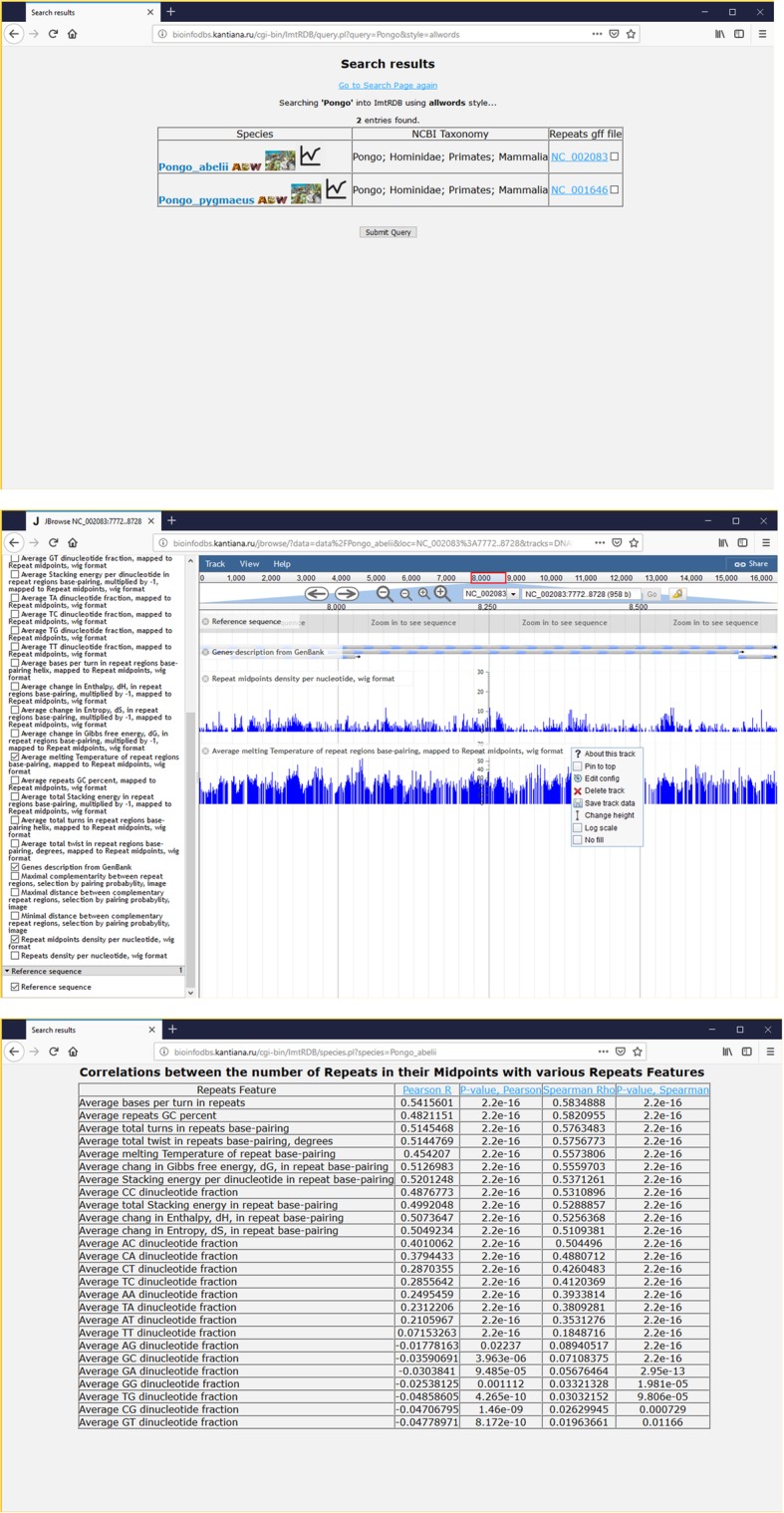
The analysis of repeat densities and repeat physico-chemical properties correlation in Pongo abelii. Upper screenshot shows ‘Search results’ page with Pongo abelii mtDNAs. Middle screenshot demonstrates the graphical comparison of Pongo abelii mtDNAs repeat densities with the melting temperatures of repeat regions base-pairing in a context of mtDNA genes. Bottom screenshot shows table that summarises the relations between Pongo abelii mtDNAs repeat densities and their physico-chemical properties

In this section we discuss several potential questions, which can be solved with the help of our database.

(I) How the repeat densities were changed along the evolution of Hominidae mtDNA? To solve this question, user enters ‘Hominidae’ in the search form on the main page. As a result the ‘Search results’ page is generated with 9 entries of various subspecies belonging to four genera: Gorilla, Homo, Pan, and Pongo (Fig. 6). After that user can select any four mtDNAs by clicking on checkboxes located in the third column of the table (Fig. 6). Clicking on ‘Submit’ button will generate tab-delimited text table with four columns representing compared mtDNAs (Fig. 6). This table is the translation of nucleotide alignment to alignment based on repeat densities (per nucleotide). This table can be visualised in any program working with spreadsheets, for example, MS Excel (Fig. 6).

(II) How the repeat densities in Pongo abelii correlate with their physico-chemical properties? To answer this question user has to enter ‘Pongo’ in the search form of the main page. The ‘Search results’ page will be generated, with 2 entries describing two various subspecies belonging to Pongo genus (Fig. 7). After that user can 1) choose graphical representation of data on repeat densities and their physico-chemical properties or 2) go to the correlation results. If user selects graphical comparison of the data he can click on the ‘Pongo_abelii’ HTML link which leads to the jBrowse page containing all data about ‘Pongo_abelii’ mtDNA. If user is interesting in the comparison of repeat densities with melting temperatures of DNA duplexes formed by repeats in a context of genes encoded by mtDNA, the user has to select three genome tracks: ‘Repeat midpoints density per nucleotide, wig format’, ‘Average melting Temperature of repeat regions base-pairing, mapped to Repeat midpoints, wig format’, and ‘Genes description from GenBank’ (Fig. 7). Mouse over and clicking on track names in the main jBrowse window allows user to download tracks data in text form. If user is interested in general data on relations between repeat densities and their physico-chemical properties in Pongo abelii he can click on graphical icon located in the first column of the ‘Search results’ page (Fig. 7).

### Abundance of repeats in mtDNA of vertebrate species

**Fig. 8:**
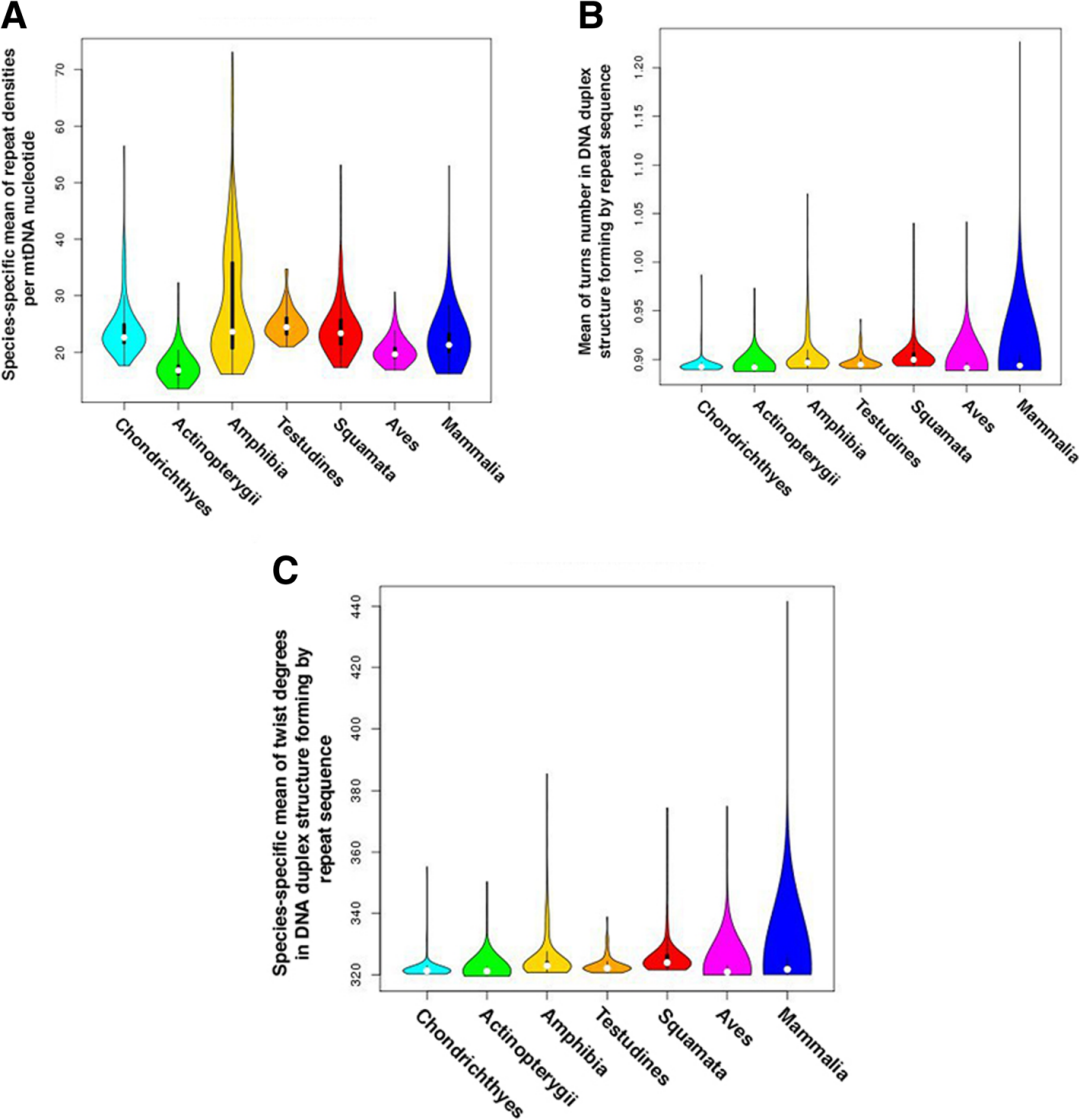
Taxa-specific repeat densities and repeat length characteristics. A, species-specific mean of repeat densities per mtDNA nucleotide; B, species-specific mean of turns number in B-DNA duplex structure forming by repeat sequence; C, species-specific mean of twist degrees in DNA duplex structure forming by repeat sequence

To compare the abundance of repeats between species we have to derive a metric, which takes into account differences in genome size as well as potential differences in repeat length. In order to do it we derived average density of repeats for each species as the following: for each nucleotide of a given genome we estimated the number of overlapped repeats and averaged it among all nucleotides of a genome. Finally, for each species we have a metric representing the number of repeats overlapping an average nucleotide (Fig. 8).

#### Mitochondrial repeats are enriched in unfolded DNA structures

Figure 8 shows that all taxa except Amphibia (higher number) and Actinopterygii (lower number) have on average 18–28 short imperfect repeats per one mtDNA nucleotide, the length of such repeats is on average 10–12 nucleotides that is equivalent to one incomplete DNA helix turn according to data shown on Fig. 8. It is of interest that on average one repeat fit to ~ 0.9 DNA turn. This fact indicates that repeats possibly concentrate in unfolded DNA regions. To analysed this question in details in each analysed species we correlates DNA turn number for repeat midpoints with the number repeats overlapping this repeat midpoints. If there is a significant positive correlation between turn number and repeats density, than repeats preferently locates in twisted regions, or, alternatively, repeats preferently locates in unfolded regions. Full results are shown in (Supplementary Tables 1 on ImtRDB site). The results indicate that regions containing repeats tended to be distributed in the twisted regions of mtDNAs, however these relations is not supported by significant Spearman Rho value (average Rho ~ 0.09). Therefore, it is most likely that the major fraction of mtDNA repeats located in unfolded structures, or, in other words, that the majority of mtDNA regions containing repeats rarely have twisted (or supertwisted) form of DNA.

#### All repeat types positively correlate with each other, but equivalent repeats correlate stronger

We checked if abundance of different types of repeats correlate with each other. We demonstrated statistically significant positive pairwise correlations between all repeat types (Table 2). The most strong correlations we observed between direct and mirror repeats as well as between inverted and complementary repeats. These repeat pairs have common features: (1) common nucleotide context, (2) common location (the same strand: direct and mirror; opposite strands: inverted and symmetrical; see Fig. 1), and (3) their short length (Fig. 8). Due to the common nature of these repeat pairs it is possible to use their similarity as an important null hypothesis, claiming that under all else equal (the same rate of origin and the same selection against or for) we expect the same number of equivalent repeats per genome. Any deviations from this equilibrium should be biologically informative and point out different strength of either mutagenesis or selection.

**Table 2:** Pairwise correlation of imperfect repeat type’s abundance and correlations with GC content, all 3716 species analyzed, Spearman Rho above diagonal, *p*-values below diagonal

	GC content, b.p.	Direct repeats, b.p.	Complementary repeats, b.p.	Mirror repeats, b.p.	Inverted repeats, b.p.
GC content		−0,2864	**−0,6965**	-0,3415	**-0,6685**
Direct repeats	< 2.2e-16		0,1247	**0,9838**	0,0919
Complementary repeats	< 2.2e-16	2.376e-14		0,1869	**0,9785**
Mirror repeats	< 2.2e-16	< 2.2e-16	< 2.2e-16		0,1426
Inverted repeats	< 2.2e-16	1.971e-08	< 2.2e-16	< 2.2e-16	

#### All repeat types negatively correlate with GC content, but inverted and complementary repeats correlate stronger

We checked if abundance of different types of repeats correlate with GC content. We observed statistically significant negative correlations between repeats abundance and their GC content (Table 2). Interestingly, the negative correlation was significantly stronger for inverted and complementary repeats (Table 2). This might be explained by the stronger negative selection against GC rich inverted and complementary repeats however additional analyses are necessary to shed a light on this observation.

**Fig. 9:**
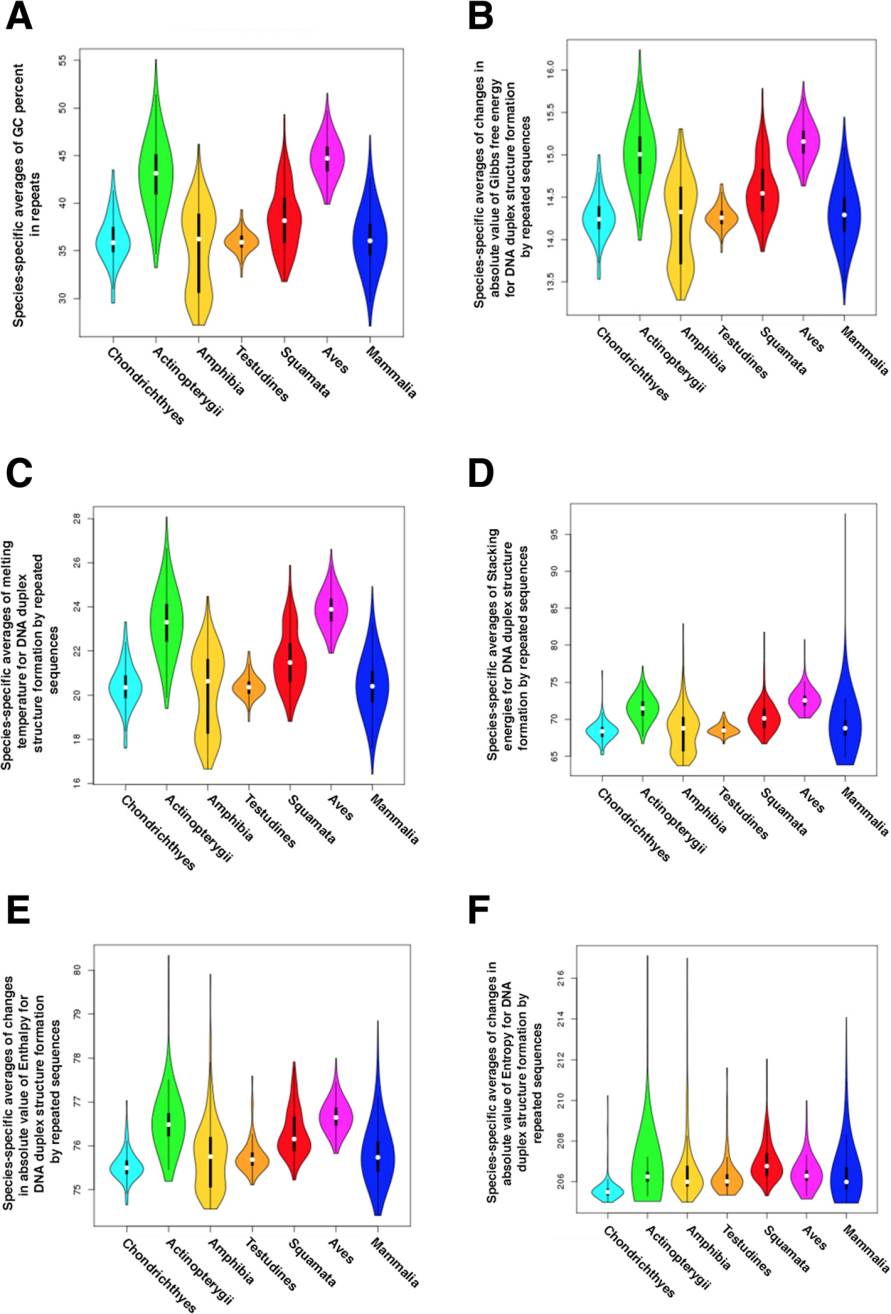
Taxa-specific repeat features related to GC content. A, species-specific averages of GC percent in repeats; B, species-specific averages of changes in absolute value of Gibbs free energy for DNA duplex structure formation by repeated sequences; C, species-specific averages of melting temperature for DNA duplex structure formation by repeated sequences; D, species-specific averages of Stacking energies for DNA duplex structure formation by repeated sequences; E, species-specific averages of changes in absolute value of Enthalpy for DNA duplex structure formation by repeated sequences; F, species-specific averages of changes in absolute value of Entropy for DNA duplex structure formation by repeated sequences

Next, taking into account potentially important role of nucleotide content in mtDNA genome evolution [71], we checked species-specific GC content in identified repeats and their relative physico-chemical features (Fig. 9). Figure 9 demonstrates that all taxa have their specific optimal GC content in repeats, for example, Actinopterygii and Aves have maximal GC content while Mammals have minimal one. Optimal taxa-specific GC content directly drives change in Gibbs free energy (dG) and melting temperature (Tm) in repeat regions base-pairing (Fig. 9). It is interesting that stacking energy of repeat regions base-pairing and base-pairing Entropy / Enthalpy (dS / dH) have significant variation in Mammals and Actinopterygii / Amphibia clades, respectively.

Next, we asked if the frequencies of complementary dinucleotide types equal in imperfect repeats located in Vertebrate mtDNAs (Table 3). This question is highly important for identification of possible selection forces acting on repeat sequences. Due to dense genome locations with nonzero dinucleotide frequencies containing in repeats (Supplementary Tables 2 on ImtRDB site) we addressed this question using genome-wide dinucleotide frequencies, however, user can perform region-based analyses of dinucleotide frequencies. We compared pairs of complementary dinucleotides observed within repeats in each analysed genome using U-test to estimate the statistical significance and Coohen’s d value to estimate the effect size. Table 3 shows average ratios, effect sizes and statistical significance level for pairs of complementary dinucleotides for all analyzed taxa (obtained by averaging species specific data). The comparison of complementary dinucleotide pairs revealed that the highest effect size was observed for CA/GT, CC/GG and AC/TG pairs (Table 3). All these ratios might be explained by positive AT skew (an excess of A versus T) and negative GC skew (deficit of G versus C) on the light chain (the chain which is always deposited in genbank) of mtDNA that is consistent with the mechanism of mtDNA replication [82, 83]. Indeed, we can see that in all these ratios (which are constructed in a way to make this ratio > 1) G and T are present only in denominators, while C and A - only in numerators. Symmetrical pairs (with identical set of nucleotides) such as TA/AT or GC/CG are not affected by the nucleotide skew and thus their ratios are expected to be close to one. Indeed TA/AT ratio doesn’t not differ from one, but interestingly, GC/CG ratio is significantly higher than one in all taxa. The reason of the excess of GC over CG dinucleotides in the light chain of mtDNA repeats of all analyzed species is worth to investigate in the future analyses. The frequency of GC is two-fold higher than the frequency of CG (Table 3). This fact is unexpectable. The reason for this issue can be rooted in the DNA-direction/strand specific regulation of mtDNA gene expression (in the case if mtDNA regions with high repeat densities can be involved in mtDNA gene expression regulation).

**Table 3:** Pairwise comparison of complementary dinucleotide pairs in mtDNA repeats

Complementary dinucleotides frequency ratio with effect size*	Chondrichthyes	Actinopteri	Amphibia	Testudines	Squamata	Aves	Mammalia
CA/GT	3.1395	2.6701	2.8525	4.9014	4.9034	4.8671	3.6059
Coohen d	**0.1601**	**0.1419**	**0.1475**	**0.234**	**0.2351**	**0.2334**	**0.188**
CC/GG	3.7044	3.0336	2.9595	3.9861	4.0513	5.3133	3.7551
Coohen d	**0.1656**	*0.1696*	**0.1283**	**0.1687**	**0.1789**	**0.2548**	**0.1588**
AC/TG	2.3030	1.9380	2.0039	3.7421	3.6465	3.4388	2.8990
Coohen d	**0.1282**	*0.1078*	**0.1086**	**0.2137**	**0.2126**	**0.1981**	**0.1657**
CT/GA	2.2609	2.0033	2	2.1529	2.3051	2.5639	2.2432
Coohen d	**0.1414**	*0.1215*	**0.1115**	**0.1232**	**0.1302**	**0.1668**	**0.1337**
AA/TT	1.1184	1.1642	1.0196	1.7303	1.6796	1.9638	1.4414
Coohen d	0.0287	0.0326	0.0055	**0.1298**	0.118	*0.1258*	0.0857
TC/AG	1.7016	1.3023	1.4356	1.4441	1.4948	1.6962	1.5327
Coohen d	**0.0901**	0.0437	0.0551	**0.0568**	*0.0601*	**0.0944**	**0.0705**
GC/CG	1.92	1.7840	1.9459	1.9579	1.7984	1.7919	1.8598
Coohen d	**0.0494**	**0.0614**	**0.0544**	**0.049**	**0.0505**	**0.0581**	**0.0493**
TA/AT	1.0345	1.1097	1.0316	1.0741	1.0871	1.0926	1.0635
Coohen d	0.0094	0.022	0.0086	0.0201	0.0208	0.0186	0.0174

*bold font, *p* < 1E-10, italic font, 1E-5 < *p* < 1E-10

## Conclusions and future directions

We observed that mitochondrial DNA imperfect repeats are generally short, frequently occurred and enriched in relaxed DNA structures.

We found strong negative correlations of repeats abundance and their GC content. This can be explained by the negative selection against GC rich repeats which is probably more pronounced in case of inverted and complementary repeats as compared to direct and mirror ones. This corresponds to common point of view that potentially deleterious effect of repeats is a function of both repeat length and GC content of the repeat.

We also observed that distribution of the majority of complementary dinucleotides on light chain of the repeated regions of mtDNA is shaped by positive AT and negative C skew, however an excess of GC over CG dinucleotides, which is strong and uniform, can not be explained by the skew and thus should be investigated additionally.

Additionally we demonstrated the strong correlation between direct and mirror repeats abundance and inverted and complementary repeats abundance. This can be explained by the similarity (equivalence) of these pairs in terms of nucleotide content.

Our database allows to answer more detail and precise questions, related to location of repeats as well as interaction between different types of repeats and interaction between repeats abundance and their phisico-chemical properties. We will regularly fill the ImtRDB database volume by addition new mtDNAs and new physico-chemical properties.

### Availability of data, database updating and support

All data and Python code of the algorithm are available on http://bioinfodbs.kantiana.ru/ImtRDB/. User can freely download the data available in ImtRDB from ‘all species listing’ pages, this will help researchers to screen imperfect repeats in mitochondrial DNA. A user-support is available to answer questions at genkvg@gmail.com and v.a.shamanskiy@gmail.com. Currently the ImtRDB is updated every year with the new mitochondrial genomes as soon as they are released in NCBI Genbank. In the future, we will try to provide additional information and will update the database each 4 months if new mitochondrial genome sequences are added in NCBI Genbank.

## Abbreviations

b.p.: base pair
dG: change in Gibbs free energy
dH: change in Enthalpy
DNAseq: DNA sequencing
dS: change in Entropy
GFF file: General feature format file
HTML link: HyperText markup language link
RNAseq: RNA sequencing
Tm: melting temperature
