# Correction to: ImtRDB: a database and software for mitochondrial imperfect interspersed repeats annotation

**DOI:** 10.1186/s12864-019-5950-4

**Published:** 2019-07-08

**Authors:** Viktor A. Shamanskiy, Valeria N. Timonina, Konstantin Yu. Popadin, Konstantin V. Gunbin

**Affiliations:** 1 Center for Mitochondrial Functional Genomics, School of Life Science, Immanuel Kant Baltic Federal University, Kaliningrad, Russia; 2 Center for Integrative Genomics, University of Lausanne, Lausanne, Switzerland; 3 Swiss Institute of Bioinformatics, Lausanne, Switzerland; 4 Center of Brain Neurobiology and Neurogenetics, Institute of Cytology and Genetics SB RAS, Novosibirsk, Russia

**Correction to: BMC Genomics**

**https://doi.org/10.1186/s12864-019-5536-1**

After publication of this supplement article [1], it was brought to our attention that the name of the first author is incorrect. The correct name should be Viktor A. Shamanskiy rather than Viktor N. Shamanskiy.
